# Post-traumatic stress disorder following intensive care for COVID-19: a single-center retrospective follow-up study

**DOI:** 10.1186/s12871-026-04256-2

**Published:** 2026-09-12

**Authors:** Carsten Zeiner, Pascal D. Schmitt, Julius J. Weise, Torben M. Rixecker, Robert Bals, Philipp M. Lepper

**Affiliations:** 1 Department of Internal Medicine V Pneumology, Allergology and Critical Care Medicine, Faculty of Medicine, Saarland University, Kirrbergerstraße 100, Homburg/Saar, 66421 Germany; 2 Faculty of Medicine, Institute for Medical Biometry, Epidemiology and Medical Informatics, Saarland University, Homburg/Saar, Germany; 3 Department of Internal Medicine, Pneumology and Intensive Care Medicine, University Hospital OWL Campus Bethel and University of Bielefeld, Bielefeld, Germany

**Keywords:** COVID-19, Post-traumatic stress disorder, Extracorporeal membrane oxygenation, Critical illness, Mental health, Risk factors, Follow-up

## Abstract

**Background:**

Severe COVID-19 frequently required prolonged intensive care, including mechanical ventilation or extracorporeal membrane oxygenation (ECMO). Survivors of such critical illness may develop post-traumatic stress disorder (PTSD).

**Methods:**

We conducted a single-center retrospective follow-up study of COVID-19 ICU survivors at Saarland University Medical Center, Germany, assessing ICD-11 trauma-related disorders using the validated International Trauma Questionnaire (ITQ) and analyzing associations with clinical factors.

**Results:**

Of 110 eligible patients, 42 (38.2%) valid datasets were obtained. The overall prevalence of ICD-11 trauma-related disorders (PTSD or CPTSD) was 23.8% (95% CI 12.1–39.5%; *n* = 10), with PTSD diagnosed in 6 participants (14.3%; 95% CI 5.4–28.5%) and CPTSD in 4 participants (9.5%; 95% CI 2.7–22.6%). No statistically significant associations were found for any of the examined variables. Exploratory subgroup analyses revealed numerically higher rates of trauma-related disorders in patients with prior psychiatric treatment (40.0% vs. 21.6%), ECMO therapy (33.3% vs. 16.7%), or renal replacement therapy (50.0% vs. 17.6%); however, these findings should be interpreted with caution given the limited sample size.

**Conclusions:**

Nearly one quarter of COVID-19 ICU survivors met the diagnostic criteria for PTSD or CPTSD, a prevalence comparable to that observed in other critically ill populations. No statistically significant associations were identified, and the observed subgroup differences should be regarded as exploratory and hypothesis-generating. The reported prevalence of PTSD/CPTSD highlights the potential value of systematic screening for trauma-related disorder and the integration of psychological support into post-ICU care. Larger prospective multicenter studies are needed to confirm these findings and further characterize potential risk factors.

**Trial registration:**

The study was reviewed and approved by the Ethics committee of the Medical Association of Saarland (Number 98/22).

**Supplementary Information:**

The online version contains supplementary material available at https://doi.org/10.1186/s12871-026-04256-2.

## Background

To date, more than 779 million cases of COVID-19 have been reported worldwide [1]. Approximately 14% of infected individuals required hospitalization, and around 32% of hospitalized patients required treatment in an intensive care unit [2, 3]. The clinical consequences of severe COVID-19 extend well beyond the acute phase of infection. While much attention has been directed toward respiratory failure, multiorgan dysfunction, and long-COVID syndromes, the psychological sequelae in survivors of critical illness have received comparatively less focus [4, 5]. Post-traumatic stress disorder (PTSD) is a recognized complication after intensive care treatment and is associated with impaired quality of life, reduced functional recovery, and increased healthcare utilization [6, 7].

Prior investigations in survivors of acute respiratory distress syndrome (ARDS) have demonstrated considerable rates of PTSD between 21 and 35% months to years after ICU discharge [8–10]. Several factors are thought to contribute to its development, including invasive interventions, prolonged sedation, delirium, and pre-existing psychiatric vulnerability [7, 11, 12]. In the context of COVID-19, these established risks may have been amplified by additional stressors such as visitor restrictions, social isolation, uncertainty regarding prognosis, and the broader societal impact of the pandemic [13, 14].

Patients requiring advanced life-support therapies, including extracorporeal membrane oxygenation (ECMO) and prolonged mechanical ventilation, represent a particularly vulnerable subgroup [15]. Despite the increasing use of these modalities during the pandemic, data on long-term psychological outcomes in this population remain limited.

The present study therefore aimed to evaluate the prevalence of ICD-11 trauma-related disorders among ICU survivors of COVID-19 and to explore potential clinical and psychosocial factors associated with these disorders.

## Methods

### Study design

Our retrospective study was conducted at the Department of Internal Medicine V – Pneumology, Allergology and Critical Care Medicine, Saarland University Medical Center, Homburg, Germany.

All patients admitted to the pulmonary intensive care unit with a confirmed diagnosis of COVID-19 between January 2020 and March 2022 were screened for eligibility. Inclusion criteria were admission or transfer to the pulmonary ICU for COVID-19 of any severity, survival to hospital discharge, and the ability to provide informed consent. In total, 110 eligible patients were identified. These patients were contacted by mail and invited to participate in a questionnaire-based survey using an expanded version of the *International Trauma Questionnaire (ITQ)*. Questionnaire responses were analyzed together with clinical and treatment data obtained from patient records. Data collection was carried out over an 18-month period between April 2022 and October 2023. To minimize selection bias, non-responders received a second mailing, and repeated follow-up phone calls were made to maximize participation. Data collection ended on October 31, 2023. Patient-reported data were collected using the validated *International Trauma Questionnaire (ITQ)*, which was extended with additional items covering demographic information, time elapsed since ICU treatment, prior psychiatric or psychological therapy, and post-discharge rehabilitation or long-COVID treatment.

**Fig. 1:**
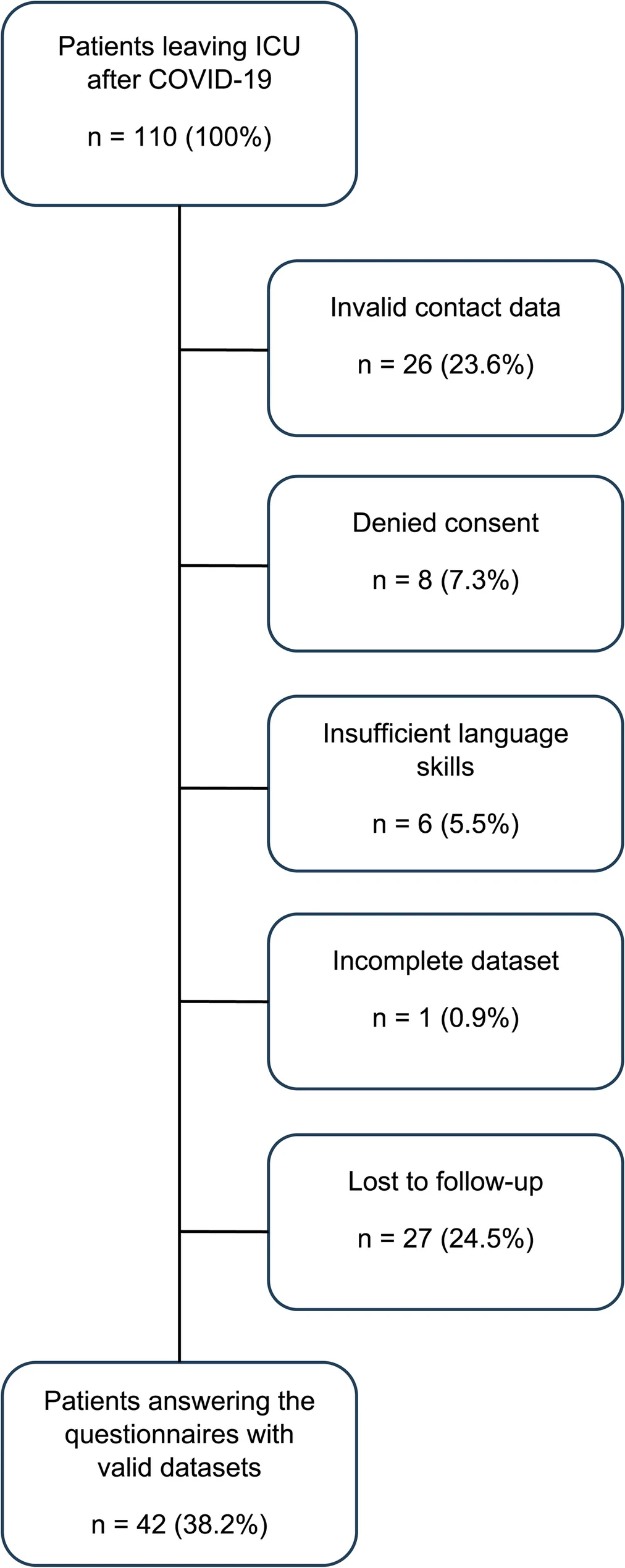
Enrollment process. Illustrates the recruitment process. Patients with invalid contact data could not be contacted because no valid postal address or telephone number was available. Patients classified as lost to follow-up were contacted but did not return the questionnaire or could not be reached despite repeated telephone contact attempts. All categories are mutually exclusive and account for all 110 eligible patients

Ultimately, 42 valid datasets were obtained from the 110 identified patients, corresponding to a response rate of 38.2%. Figure 1 illustrates the recruitment process.

No formal sample size calculation was performed because this was an exploratory observational study, and all elegible ICU survivors treated during the predefined study period were invited to participate.

The study was reviewed and approved by the Ethics committee of the Medical Association of Saarland (Number 98/22).

This manuscript was prepared in accordance with the Strengthening the Reporting of Observational Studies in Epidemiology (STROBE) Statement [16].

### International Trauma Questionnaire (ITQ) and assessment of PTSD and CPTSD

The International Trauma Questionnaire (ITQ) is a self-report instrument initially developed in English and later validated in German for the assessment of post-traumatic stress disorder (PTSD) and complex PTSD according to the 11th revision of the International Classification of Diseases (ICD-11) [17–19].

It comprises two sections evaluating symptoms of PTSD and complex PTSD (CPTSD) as defined by ICD-11. Each item is rated on a 5-point Likert scale (0 = “not at all” to 4 = “extremely”). Part 1 addresses re-experiencing, avoidance, sense of current threat, and functional impairment related to PTSD symptoms during the past month. Part 2 includes items assessing affective dysregulation, negative self-concept, disturbed relationships, and associated functional impairment as indicators of CPTSD.

For this study, additional questions were included before and after the ITQ items to capture relevant clinical and psychosocial factors such as timing of the ICU stay, pre-existing psychiatric care, subsequent rehabilitation, and ongoing psychological or psychiatric therapy.

PTSD and CPTSD were diagnosed according to the scoring algorithm of the *International Trauma Questionnaire (ITQ)*. A detailed description is available in Additional file 1.

### Statistical analysis

For the statistical analyses, participants meeting the diagnostic criteria for either PTSD or CPTSD were combined into a single category of ICD-11 trauma-related disorders.

Medication exposure was analyzed as a binary variable (administration at any time during the ICU stay: yes/no). Medication categories were not mutually exclusive.

Durations of organ support therapies (ECMO, invasive mechanical ventilation, NIV, and HFNC) were analyzed only among patients who received the respective therapy.

Statistical analyses were performed using IBM SPSS Statistics version 28.0.0.1 (IBM Corp., Armonk, NY, USA). Continuous variables are presented as mean ± standard deviation (SD) or median (interquartile range [IQR]), as appropriate, and categorical variables as frequencies and percentages. Group comparisons between patients with and without ICD-11 trauma-related disorders (PTSD/CPTSD) were performed using the independent-samples t test or Mann–Whitney U test for continuous variables, depending on the distribution of the data (assessed using the Shapiro–Wilk test), and the Chi² test or Fisher’s exact test for categorical variables, as appropriate. Associations between clinical variables and trauma-related disorders were explored using univariable binary logistic regression, with results reported as odds ratios (ORs) and 95% confidence intervals (CIs). Statistical significance was defined as a two-sided *p* value < 0.05.

## Results

In total, 42 patients were included in the analysis, of whom 30 (71.4%) were male and 12 (28.6%) female. Patient characteristics are shown in Table 1.

**Table 1:** Demographic and clinical characteristics of patients with or without PTSD of the study cohort

Demographic and clinical characteristics		Whole sample	No PTSD	PTSD	*p*-value^‡^
All patients, *n (%)*		42 (100.0)	32 (76.2)	10 (23.8)	
Age *years*, (mean, SD)		61.2 ± 11.0	59.8 ± 11.8	65.6 ± 6.4	0.145^c^
Sex	male	30 (71.4)	23 (76.7)	7 (23.3)	1.000^a^
	female	12 (28.6)	9 (75.0)	3 (25.0)	
Time until survey	< 6 months	1	1 (100.0)	0 (0.0)	0.262^a^
	6–12 months	7	4 (57.1)	3 (42.9)	
	1–5 years	34	27 (79.4)	7 (20.6)	
Prior psychological therapy	yes	5 (11.9)	3 (60.0)	2 (40.0)	0.577^a^
	no	37 (88.1)	29 (78.4)	8 (21.6)	
Post ICU psychological therapy	yes	6 (14.3)	5 (83.3)	1 (16.7)	1.000^a^
	no	36 (85.7)	27 (75.0)	9 (25.0)	
Post ICU rehabilitation	yes	26 (61.9)	18 (69.2)	8 (30.8)	0.270^a^
	no	16 (38.1)	14 (87.5)	2 (12.5)	
„Long covid“ therapy	yes	12 (28.6)	9 (75.0)	3 (25.0)	1.000^a^
	no	30 (71.4)	23 (76.6)	7 (23.3)	
ECMO	yes	18 (42.9)	12 (66.7)	6 (33.3)	0.281^a^
	no	24 (57.1)	20 (83.3)	4 (16.7)	
ECMO *days* (median, IQR)		28.0 (19.3–41.5)^#^	30.0 (21.8–49.8)	22.0 (12.0-33.3)	0.213^d^
RRT	yes	8 (19.0)	4 (50.0)	4 (50.0)	0.075^a^
	no	34 (81.0)	28 (82.4)	6 (17.6)	
Tracheostomy	yes	23 (54.8)	17 (73.9)	6 (26.1)	1.000^a^
	no	19 (45.2)	15 (78.9)	4 (21.1)	
SAE	yes	17 (40.5)	14 (82.4)	3 (17.6)	0.490^a^
	no	25	18 (72.0)	7 (28.0)	
ICU stay *days* (median, IQR)		29.0 (5.8–50.3)	28.0 (4.3–55.0)	32.0 (9.0-50.3)	0.673^d^
Invasive ventilation *days* (median, IQR)		36.5 (26.0–54.0) ^#^	36.5 (25.5–54.5)	36.5 (25.3–49.5)	0.871^d^
Non-invasive ventilation *days* (median, IQR)		10.0 (5.0–15.0) ^#^	9.0 (3.0–14.0)	14.0 (*n* = 2)	†
HFNC *days* (median, IQR)		5.0 (2.5-9.0) ^#^	8.5 (4.25–24.3)	4.5 (2.0–6.0)	0.132^d^
RASS <= -2 *days* (median, IQR)		2.0 (0.0-17.5)	2.0 (0.0-18.3)	4.0 (0.0-16.5)	0.681^d^
Comorbidities	cardiovascular	23 (54.8)	16 (69.6)	7 (30.4)	0.305^a^
	pulmonal	8 (19.0)	5 (62.5)	3 (37.5)	0.369^a^
	metabolic	16 (38.1)	11 (68.8)	5 (31.3)	0.465^a^
	neoplastic	3 (7.1)	3 (100.0)	0 (0.0)	1.000^a^
Anesthetics	Opioids	26 (63.4)	20 (76.9)	6 (23.1)	1.000^a^
	Propofol	22 (53.7)	17 (77.3)	5 (22.7)	1.000^a^
	Benzodiazepines	15 (36.6)	12 (80.0)	3 (20.0)	1.000^a^
	Clonidine	21 (51.2)	17 (81.0)	4 (19.0)	0.719^a^
	Ketamine	2 (4.9)	2 (100.0)	0 (0.0)	1.000^a^

Data are shown as numbers n (%),mean ± SD or median (IQR). Abbreviations: PTSD, post-traumatic stress disorder; ICU, intensive care unit; ECMO, extracorporeal membrane oxygenation; RRT, renal replacement therapy; SAE, severe adverse events; HFNC, high-flow nasal cannula; RASS, Richmond Agitation Sedation Scale

^a^ Fisher’s exact test if one of the expected cell frequencies was < 5

^b^ Chi2 test if all the expected cell frequencies were ≥ 5

^c^ T-test for comparison of means

^d^ Mann-Whitney-U-Test;

† Statistical comparison was not performed because only two patients in the TRD group received non-invasive ventilation

^**‡**^ Comparison between patients with PTSD and without PTSD

^#^ Durations of organ support therapies were analyzed only among patients who received the respective therapy

Overall, 10 of 42 patients (23.8%; 95% CI 12.1–39.5%) met diagnostic criteria for an ICD-11 trauma-related disorder (PTSD or CPTSD) according to the International Trauma Questionnaire (ITQ). Specifically, PTSD was diagnosed in 6 patients (14.3%; 95% CI 5.4–28.5%) and CPTSD in 4 patients (9.5%; 95% CI 2.7–22.6%). No significant associations were observed between the presence of trauma-related disorder and any of the examined variables, including gender or age. PTSD/CPTSD occurred in 23.3% (*n* = 7) of male and 25.0% (*n* = 3) of female patients (*p* = 1.000).

The mean (± SD) age at the time of data collection was 65.6 ± 6.4 years (range 55–74) for patients with a trauma-related disorder and 59.8 ± 11.8 (range 34–82) for patients without PTSD/CPTSD, not differing significantly (*p* = 0.145).

When stratified by the interval between intensive care treatment and follow-up, the observed prevalence of PTSD/CPTSD was 0% (*n* = 0/1, < 6 months), 42.9% (*n* = 3/7, 6–12 months) and 20.6% (*n* = 7/34, 1–5 years); however, these differences were not statistically significant (*p* = 0.262).

Pre-existing psychiatric or psychotherapeutic treatment was reported by five patients (11.9%), and these individuals showed a numerically higher rate of PTSD and CPTSD compared to those without such a history (40.0% vs. 21.6%, *p* = 0.577). After ICU therapy, six patients (14.3%) reported receiving psychiatric or psychotherapeutic support; PTSD/CPTSD was numerically less frequent among these patients than among those who did not receive such therapy (16.7% vs. 25.0%, *p* = 1.000).

Twenty-six patients (61.9%) underwent post-acute rehabilitation. In exploratory analyses, the proportion of pacients with PTSD/CPTSD was numerically higher among patients who underwent rehabilitation than among those who did not (30.8 vs. 12.5%, *p* = 0.270) Twelve patients (28.6%) received treatment for long COVID, with no significant difference in PTSD/CPTSD prevalence compared to those without such therapy (25.0% vs. 23.3%, *p* = 1.000). 

Eighteen patients (42.9%) had required extracorporeal membrane oxygenation (ECMO). In exploratory analyses, the proportion of patients with PTSD and CPTSD was numerically higher among patients treated with ECMO than among those without ECMO (33.3 vs. 16.7%, *p* = 0.281). The median ECMO duration was 28.0 days (IQR 19.3–41.5). ECMO duration did not differ significantly between patients with and without a trauma-related disorder (22.0 [12.0–33.3] vs. 30.0 [21.8–49.8], *p* = 0.213). Consistently, in univariable logistic regression analysis, ECMO duration was not significantly associated with PTSD/CPTSD (OR = 0.958, 95% CI 0.892–1.028; *p* = 0.231). Eight patients (19.0%) had required renal replacement therapy. The proportion of patients with PTSD/CPTSD was numerically higher among those requiring renal replacement therapy than among those who did not (50.0 vs. 17.6%, *p* = 0.075); however, this difference was not statistically significant.

Tracheostomy was performed in 23 patients (54.8%). PTSD/CPTSD was numerically more frequent among tracheotomized than non-tracheotomized patients (26.1 vs. 21.1%, *p* = 1.000). Severe adverse events such as deep vein thrombosis, pulmonary embolism, pneumothorax, hemothorax, or resuscitation occurred in 17 patients (40.5%), and PTSD/CPTSD was descriptively less frequent in this group (17.6%) compared with patients without severe events (28.0%, *p* = 0.490).

The median ICU length of stay was 29.0 days (IQR 5.8–50.3). ICU length of stay did not differ significantly between patients with and without trauma-related disorder (32.0 [9.0–50.3] vs. 28.0 [4.3–55.0] days, *p* = 0.673).Consistently, in univariable logistic regression analysis, ICU length of stay was not associated with PTSD/CPTSD (OR = 0.999, 95% CI 0.974–1.025; *p* = 0.951). Patients received different forms of respiratory support during their ICU stay, including oxygen therapy (*n* = 7, 16.7%), HFNC (*n* = 12, 28.6%), NIV (*n* = 13, 31.0%), and invasive mechanical ventilation (*n* = 24, 57.1%). As respiratory support was frequently escalated during the course of treatment, these categories were not mutually exclusive. The median duration of invasive mechanical ventilation was 36.5 days (IQR 26.0–54.0). Duration of invasive mechanical ventilation did not differ significantly between patients with and without PTSD/CPTSD (36.5 [25.3–49.5] vs. 36.5 [25.5–54.5] days, *p* = 0.871). In univariable logistic regression analysis, duration of invasive mechanical ventilation was not associated with PTSD/CPTSD (OR = 0.993, 95% CI 0.947–1.040; *p* = 0.754). Thirteen patients required non-invasive ventilation, including two patients with a trauma-related disorder. The median duration of non-invasive ventilation was 9.0 days (IQR 3.0–14.0) in patients without PTSD/CPTSD and 14.0 days in the two patients with PTSD/CPTSD. Owing to the very small number of PTSD/CPTSD patients requiring non-invasive ventilation, no formal statistical comparison of NIV duration between groups was performed. Among patients receiving HFNC therapy, the median duration of HFNC was 8.5 days (IQR 4.25–24.3) in patients without PTSD/CPTSD and 4.5 days (IQR 2.0–6.0) in patients with PTSD/CPTSD. HFNC duration did not differ significantly between the two groups (*p* = 0.132). In univariable logistic regression analysis, HFNC duration was not associated with a trauma-related disorder (OR = 0.776, 95% CI 0.512–1.175; *p* = 0.231).The number of days with a Richmond Agitation-Sedation Scale (RASS) score ≤ − 2 ranged from 0 to 48 (median 2.0 [0.0–17.5]) ) and did not differ significantly between the two groups (*p* = 0.681). Cardiovascular comorbidities were present in 23 patients (54.8%). Although not statistically significant, numerically higher proportions of PTSD/CPTSD were observed among these patients (30.4% vs. 15.8%, *p* = 0.305). Pulmonary comorbidities were found in eight patients (19.0%). PTSD/CPTSD was numerically higher among these patients than among those without pulmonary comorbidities (37.5 vs. 20.6%, *p* = 0.369). Sixteen patients (38.1%) had metabolic disorders (including obesity, type 2 diabetes mellitus, chronic kidney disease and thyreoid disorders). Similarly, these showed a numerically higher rate of PTSD/CPTSD (31.3% vs. 19.2%, *p* = 0.465). Three patients (7.1%) had neoplastic disease and none met the diagnostic criteria for a trauma-related disorder.

Medication data were available for 41 of the 42 patients. Medication records for one patient were unavailable and could therefore not be included in the analysis. The most frequently used drugs during ICU treatment were opioids (63.4%), propofol (53.7%), clonidine (51.2%), and benzodiazepines (36.6%), while ketamine was used in only two patients (4.9%). No significant associations were observed between any medication and PTSD/CPTSD. Numerically, a trauma-related disorder was present in 23.1% of patients receiving opioids, 22.7% receiving propofol, 20.0% receiving benzodiazepines, and 19.0% receiving clonidine. None of the patients receiving ketamine met the diagnostic criteria fpr PTSD/CPTSD.

## Discussion

Among ICU-treated patients in our cohort, 23.8% met criteria for an ICD-11 trauma-related disorder, clearly exceeding rates reported in the general population. A 2025 systematic review reported a pooled prevalence of 2% in non-war-exposed populations and 16% in war-affected or economically less developed regions [20] while a representative German sample showed a prevalence of 1.5% [21].

Our findings are consistent with previous studies of COVID-19 ICU survivors. An Italian study reported a PTSD prevalence of 23.9% among survivors of COVID-19-related ARDS [22], and another study reported a prevalence of 30.2% [23]. Moreover, our results are comparable to findings in non-COVID ICU populations. A large prospective observational study conducted in the United Kingdom prior to the pandemic demonstrated that 22% of ICU survivors met criteria for PTSD three months after discharge, with an additional 7% developing PTSD by 12 months [10]. Similarly, a 2015 meta-analysis reported a pooled prevalence of approximately 20% [7], and a 2019 systematic review found a point prevalence of 19.83%, with the highest rates observed 12 months after ICU discharge [24]. Taken together, these findings suggest that PTSD prevalence among COVID-19 ICU survivors is broadly comparable to that reported in other critically ill populations.

The elevated prevalence of a trauma-related disorder in ICU-treated patients compared with the general population is likely multifactorial. Survivors of intensive care frequently experience long-term physical impairments, neuropsychiatric sequelae, and reduced quality of life [6]. This has been demonstrated particularly in survivors of ARDS, in whom Herridge et al. showed persistent physical impairment and reduced quality of life one year after critical illness, with substantial functional disability still present five years later [25, 26]. These landmark studies highlighted that recovery after severe respiratory failure is often incomplete and extends far beyond hospital discharge. Similarly, Schmidt et al. reported that survivors of severe ARDS treated with ECMO may experience impaired long-term quality of life and functional outcomes despite acceptable long-term survival [27]. These findings support the concept that survivors of severe critical illness remain at risk for persistent physical and psychological sequelae, including PTSD [15, 27]. Patients’ recollections of invasive procedures are often characterized by intense distress, and emotional responses such as fear, frustration, or depressive symptoms have been linked to subsequent psychological morbidity [28, 29]. Delusional memories, particularly in the absence of factual recollection of the ICU stay, have also been associated with increased PTSD incidence [30].

Additional contributing factors may include prolonged immobilization, exposure to an unfamiliar environment, and the perception of life-threatening illness. Both objective factors (e.g., length of ICU or hospital stay) and subjective evaluation of the ICU experience have been associated with adverse emotional outcomes [31]. In addition, strict visitation restrictions during the pandemic resulted in pronounced social isolation. The absence of familiar emotional support may have impaired emotional processing and increased feelings of helplessness. Social support is a well-established protective factor against PTSD, and its absence represents a significant risk factor [13]. Furthermore, the pandemic itself may be conceptualized as a form of collective trauma, characterized by continuous media reporting, rising mortality rates, and strain on healthcare systems.

In our cohort, the highest proportion of PTSD/CPTSD was observed among patients assessed 6–12 months after ICU treatment. However, given the small number of patients in this subgroup and the cross-sectional design, this observation should be considered descriptive only. Although previous studies have suggested that PTSD symptoms may emerge or become more apparent over time [24, 32], the present study was not designed to evaluate symptom trajectories or draw conclusions regarding the temporal course of trauma-related disorders. Furthermore, the lack of structured follow-up at predefined intervals limited the assessment to PTSD/CPTSD symptoms at a single point in time, resulting in heterogeneous follow-up intervals and small subgroup sizes.

In our exploratory analyses, patients with pre-existing psychiatric conditions showed a numerically higher prevalence of PTSD/CPTSD (40.0 vs. 21.6%). Although this difference was not statistically significant, it is consistent with previous reports: Davydow et al. identified pre-existing psychiatric illness as a risk factor for PTSD after critical illness [11], and similar associations were described in COVID-19 patients by Tarsitani et al. [33]. Reduced emotional and cognitive resources [34], maladaptive cognitive processing as described by Ehlers et al. [35], and neurobiological alterations involving the amygdala and prefrontal cortex [36] may contribute to increased susceptibility.

Regarding organ replacement therapies, ECMO-treated patients showed a numerically higher prevalence of trauma-related disorder (33.3% versus 16.7%), although this difference did not reach statistical significance. These findings are consistent with prior reports demonstrating PTSD prevalence rates of 29.4% in VA-ECMO patients [37] and 30.8% in another ECMO cohort [15]. Although our results are consistent with these reports, they should be interpreted as exploratory given the limited size and lack of statistical significance. Potential mechanisms discussed in the literature include prolonged immobilization, perceived life threat, deep sedation [38], and delirium-related mechanisms [39].

A numerically higher prevalence of PTSD/CPTSD was also observed in patients receiving renal replacement therapy (50.0 vs. 17.6%), similar to that seen in patients undergoing ECMO; however, this was not statistically significant either. Although specific data on ICU-related renal replacement therapy and PTSD remain scarce, a lifetime PTSD prevalence of 17% has been reported among long-term hemodialysis patients [40], and declining renal function has been associated with higher PTSD rates [41]. The need for organ replacement therapy may serve as a marker of disease severity, which has been linked to an increased risk of long-term psychological sequelae [42]. In a 2020 ECMO cohort, nearly half of patients reported anxiety, depression, or PTSD symptoms, and only one third felt that these issues were adequately addressed after discharge [12].

Patients undergoing post-acute rehabilitation exhibited a numerically higher PTSD/CPTSD prevalence (30.8%) compared with those who did not undergo rehabilitation (12.5%). Whether this reflects greater disease severity or a more intensive confrontation with traumatic experiences during structured rehabilitation cannot be determined from our study. The integration of psychological support into rehabilitation programs has been emphasized in previous literature [43].

Several limitations should be considered. Although not uncommon for this type of survey, the moderate response rate (38.2%) entails a relevant risk of selection bias, as it remains unclear whether patients with greater or lesser psychological burden were more likely to participate. Consequently, the reported prevalence of PTSD/CPTSD may either overestimulate or underestimulate the true prevalence in ICU survivors, thereby limiting the generalizability of our findings. The relatively high proportion of ECMO-treated patients reflects the role of our institution as a tertiary referral center for severe respiratory failure. Consequently, the study population may not be representative of general ICU populations, which may limit the generalizability of our findings. Furthermore, patients with insufficient German-language skills were excluded because the questionnaire was only provided in German. This may have reduced the representativeness of the study population, as our cohort included patients from neighboring countries as well as individuals with a migration background. Consequently, the reported prevalence may not be fully representative of all ICU survivors with COVID-19. The small sample size, combined with the large number of variables examined, limited statistical power and reduced the reliability of subgroup analyses. Given the exploratory nature of this study, numerous subgroup comparisions were performed without adjustment for multiple testing.Therefore, the findings from these analyses should be interpreted with caution and regarded as hypothesis-generating rather than confirmatory. Moreover, the retrospective cross-sectional design and the heterogeneous intervals between ICU discharge and follow-up precluded conclusions regarding the temporal course of PTSD/CPTSD symptoms. As symptoms were assessed only once using the ITQ, the study could not distinguish between symptom onset, persistence, or remission over time. In addition, the absence of systematic follow-up at predefined time points may have introduced bias, as participants may have completed the questionnaire before symptoms emerged or after they had resolved. Finally, although the modified ITQ instructed patients to relate their responses to their ICU treatment, we did not assess exposure to other potentially traumatic or stressful life events occurring between ICU discharge and follow-up. Consequently, the observed PTSD/CPTSD symptoms cannot be attributed exclusively to the ICU stay or COVID-19 illness.

## Conclusion

In summary, approximately one quarter of patients treated for severe COVID-19 in our ICU met the diagnostic criteria for an ICD-11 trauma-related disorder, a prevalence comparable to that observed in other critically ill populations but markedly higher than in the general population. No statistically significant associations were found between trauma-related disorder and any of the examined variables, reflecting the limited statistical power of this exploratory study. Exploratory subgroup analyses revealed numerically higher rates of PTSD/CPTSD in patients receiving ECMO therapy, renal replacement therapy, and among those with pre-existing psychiatric conditions; however, these findings should be interpreted with caution given the limited sample size.

Despite these limitations, our results underscore that psychological health represents a critical component of long-term recovery after severe COVID-19. They support systematic screening for trauma-related disorder and the proactive integration of psychological follow-up into post-ICU and rehabilitation care. Increased awareness among healthcare providers is essential to ensure early identification and timely intervention for affected patients, as symptoms may emerge months after ICU discharge.

Future prospective, multicenter studies with larger patient cohorts are needed to validate these findings and better characterize temporal dynamics and potential implications for patient care.

## Supplementary Information

Additional file 1. International Trauma Questionnaire used in this study. Diagnostic scoring for PTSD and CPTSD.

Additional file 2.

## Data Availability

The datasets used and analyzed during the current study are available from the corresponding author on reasonable request.

## Abbreviations

CI: Confidence interval
COVID-19: Coronavirus disease 2019
CPTSD: Complex post-traumatic stress disorder
ECMO: Extracorporeal membrane oxygenation
HFNC: High-flow nasal cannula
ICD: International Classification of Diseases
ICU: Intensive care unit
IQR: Interquartil range
ITQ: International Trauma Questionnaire
OR: Odds ratio
PTSD: Post-traumatic stress disorder
RASS: Richmond Agitation Sedation Scale
RRT: Renal replacement therapy
SAE: Severe adverse events
SD: Standard deviation
