# Supplementary material for: Post-traumatic stress disorder following intensive care for COVID-19: a single-center retrospective follow-up study
Source: BMC Anesthesiol. 2026 Sep 12;26:561. doi: 10.1186/s12871-026-04256-2 (PMC13570528; doi:10.1186/s12871-026-04256-2)
Supplement: Supplementary file 1 [file 12871_2026_4256_MOESM1_ESM.docx]

**International Trauma Questionnaire (ITQ) *(modified for use in this study)***

When did your inpatient treatment for COVID-19 take place?

☐ less than 6 months ago  ☐ 6 to 12 months ago  ☐ 1 to 5 years ago

Prior to your COVID-19 illness, did you receive psychiatric and/or psychological treatment (including completed courses of therapy)?

☐ yes  ☐ no

The following section lists problems and symptoms that may occur as consequences of traumatic or distressing life experiences. Please answer the questions with reference to your experiences during your recent intensive care treatment for your COVID-19 illness.

Below are a number of problems that people sometimes report in response to traumatic or stressful life events. Please read each item carefully, then circle one of the numbers to the right to indicate how much you have been bothered by that problem in the past month.

|  | ***Not***  ***at all*** | ***A little***  ***bit*** | ***Moderately*** | ***Quite***  ***a bit*** | ***Extremely*** |
| --- | --- | --- | --- | --- | --- |
| P1. Having upsetting dreams that replay part of the experience or are clearly related to the experience? | 0 | 1 | 2 | 3 | 4 |
| P2. Having powerful images or memories that sometimes come into your mind in which you feel the experience is happening again in the here and now? | 0 | 1 | 2 | 3 | 4 |
| P3. Avoiding internal reminders of the experience (for example, thoughts, feelings, or physical sensations)? | 0 | 1 | 2 | 3 | 4 |
| P4. Avoiding external reminders of the experience (for example, people, places, conversations, objects, activities, or situations)? | 0 | 1 | 2 | 3 | 4 |
| P5. Being “super-alert”, watchful, or on guard? | 0 | 1 | 2 | 3 | 4 |
| P6. Feeling jumpy or easily startled? | 0 | 1 | 2 | 3 | 4 |
| ***In the past month have the above problems:*** | | | | | |
| P7. Affected your relationships or social life? | 0 | 1 | 2 | 3 | 4 |
| P8. Affected your work or ability to work? | 0 | 1 | 2 | 3 | 4 |
| P9. Affected any other important part of your life such as parenting, or school or college work, or other important activities? | 0 | 1 | 2 | 3 | 4 |

Below are problems that people who have had stressful or traumatic events sometimes experience. The questions refer to ways you typically feel, ways you typically think about yourself and ways you typically relate to others. Answer the following thinking about how true each statement is of you.

| ***How true is this of you?*** | ***Not***  ***at all*** | ***A little***  ***bit*** | | ***Moderately*** | | | ***Quite***  ***a bit*** | ***Extremely*** | |
| --- | --- | --- | --- | --- | --- | --- | --- | --- | --- |
| C1. When I am upset, it takes me a long time to calm down. | 0 | | 1 | | 2 | 3 | | | 4 |
| C2. I feel numb or emotionally shut down. | 0 | | 1 | | 2 | 3 | | | 4 |
| C3. I feel like a failure. | 0 | | 1 | | 2 | 3 | | | 4 |
| C4. I feel worthless. | 0 | | 1 | | 2 | 3 | | | 4 |
| C5. I feel distant or cut off from people. | 0 | | 1 | | 2 | 3 | | | 4 |
| C6. I find it hard to stay emotionally close to people. | 0 | | 1 | | 2 | 3 | | | 4 |

***In the past month, have the above problems in emotions, in beliefs about yourself and in relationships:***

| C7. Created concern or distress about your relationships or social life? | 0 | 1 | 2 | 3 | 4 |
| --- | --- | --- | --- | --- | --- |
| C8. Affected your work or ability to work? | 0 | 1 | 2 | 3 | 4 |
| C9. Affected any other important parts of your life such as parenting, or school or college work, or other important activities? | 0 | 1 | 2 | 3 | 4 |

Following discharge, did you undergo a course of rehabilitative treatment (post-acute rehabilitation) related to your COVID-19 illness?

☐ yes  ☐ no

Following discharge, did you receive treatment for persistent symptoms consistent with Long COVID?

☐ yes  ☐ no

Since your discharge, have you received psychiatric and/or psychological treatment, or are you currently undergoing such treatment?

☐ yes  ☐ no

**Diagnostic scoring for PTSD and CPTSD**

**PTSD**

If P1 or P2 ≥ 2 criteria for Re-experiencing in the here and now (Re_dx) met

If P3 or P4 ≥ 2 criteria for Avoidance (Av_dx) met

If P5 or P6 ≥ 2 criteria for Sense of current threat (Th_dx) met

AND

At least one of P7, P8, or P9 ≥ 2 meets criteria for PTSD functional impairment (PTSDFI).

If criteria for ‘Re_dx’ AND ‘Av_dx’ AND ‘Th_dx’ AND ‘PTSDFI’ are met, the criteria for PTSD are met.

**CPTSD**

If C1 or C2 ≥ 2 criteria for Affective dysregulation (AD_dx) met

If C3 or C4 ≥ 2 criteria for Negative self-concept (NSC_dx) met

If C5 or C6 ≥ 2 criteria for Disturbances in relationships (DR_dx) met

AND

At least one of C7, C8, or C9 ≥ 2 meets criteria for DSO functional impairment (DSOFI)

If criteria for ‘AD_dx’ AND ‘NSC_dx’ AND ‘DR_dx’, and ‘DSOFI’ are met, the criteria for DSO are met.

PTSD is diagnosed if the criteria for PTSD are met but NOT for DSO.

CPTSD is diagnosed if the criteria for PTSD are met AND criteria for DSO are met.

Not meeting the criteria for PTSD or meeting only the criteria for DSO results in no diagnosis.
